# Clinical outcomes of intravascular ultrasound-guided drug-coated balloon angioplasty for acute coronary syndrome: A single-center retrospective cohort study

**DOI:** 10.1097/MD.0000000000050803

**Published:** 2026-10-02

**Authors:** Wei Wen, Dongdong Yu, Haibo Chen

**Affiliations:** a Shantou University Medical College, Shantou, Guangdong, China; b Department of Cardiology, The First Affiliated Hospital of Shenzhen University, Shenzhen, Guangdong, China.

**Keywords:** acute coronary syndrome, drug-coated balloon, intravascular ultrasound, major adverse cardiovascular events, precision intervention

## Abstract

Although drug-coated balloons (DCBs) are increasingly used in acute coronary syndrome (ACS), evidence regarding mid-term clinical outcomes after DCB angioplasty remains limited. Real-world data evaluating whether intravascular ultrasound (IVUS)-guided lesion preparation is associated with improved outcomes compared with angiography-guided intervention are scarce, particularly in patients with complex coronary lesions. This study compares clinical outcomes associated with IVUS versus coronary angiography (CAG) guidance for DCB angioplasty in patients with ACS, focusing on major adverse cardiovascular events (MACE) and net adverse clinical events (NACE) within the prespecified 24-month observation window. This retrospective study included 229 patients with ACS treated with DCB angioplasty between October 2017 and June 2025. Patients were categorized into IVUS-guided (n = 122) and CAG-guided (n = 107) groups according to the imaging strategy used during the procedure. Inverse probability of treatment weighting (IPTW) was applied to adjust for baseline confounders. The primary endpoints were MACE and NACE occurring within the prespecified 24-month observation window after DCB angioplasty. Secondary outcomes included individual clinical events, procedural characteristics, length of stay, and biochemical parameters assessed at predefined follow-up time points. After IPTW adjustment, baseline characteristics were balanced between groups. Procedural parameters, including DCB diameter, number of DCBs deployed, and bailout stenting rate, were comparable (all *P* > .05). Within the prespecified 24-month observation window, MACE occurred less frequently in the IVUS group than in the CAG group (8.2% vs 17.8%; IPTW *P* = .038). Major bleeding was also lower in the IVUS group (1.6% vs 11.2%; IPTW *P *= .015), contributing to a lower incidence of NACE (9.8% vs 26.2%; IPTW *P* = .001). IVUS guidance was additionally associated with a shorter hospital stay (4.2 vs 5.8 days; *P* < .001), while biochemical parameters remained comparable during follow-up. In patients with ACS treated with DCB, IVUS‑guided angioplasty was associated with significantly lower MACE and improved NACE outcomes during follow-up compared with angiography‑guided therapy alone. This benefit was largely attributable to an improved safety profile (e.g., fewer bleeding events). The associations remained consistent across subgroups, suggesting that IVUS‑guided lesion preparation may offer clinical value in this population and warrants further investigation.

## 1. Introduction

Percutaneous coronary intervention (PCI) is increasingly shifting from permanent mechanical scaffolding toward a “leave nothing behind” strategy, aimed at preserving physiological vasomotion and anatomical integrity.^[1]^ Drug-coated balloons (DCBs) have emerged as an important component of this paradigm. They deliver antiproliferative drugs (e.g., paclitaxel) to the vascular wall through a lipophilic carrier matrix, effectively suppressing neointimal hyperplasia. This approach circumvents the long-term inflammatory and vasomotor limitations associated with permanent metallic implants, positioning DCB angioplasty as an alternative therapeutic strategy to conventional stenting.^[2]^

Although the clinical applications of DCBs are expanding beyond in-stent restenosis (ISR) and small vessels to include de novo large vessel lesions, their routine application in acute coronary syndrome (ACS) remains actively debated. A 2025 Japanese multicenter registry reported non-inferiority of DCBs compared to drug-eluting stents (DES) for de novo ACS lesions.^[3]^ Conversely, 3-year outcomes from the REC-CAGEFREE I trial demonstrated the inferiority of standalone DCB against DES in unselected de novo populations, driven predominantly by elevated rates of target lesion revascularization (TLR).^[4]^

Emerging data attribute this efficacy gap to suboptimal lesion preparation. Conventional angiography cannot adequately visualize the complex ACS morphology – plaque rupture, lipid cores, and thrombus – nor accurately measure reference vessel diameters or detect post‑dilation intimal tears.^[5]^ Consequently, reliance on angiography alone may contribute to inappropriate DCB sizing, suboptimal drug delivery, and failure to identify clinically relevant dissections or intramural hematomas. These procedural limitations may be associated with increased risks of acute vessel closure and restenosis.^[6]^

Intravascular ultrasound (IVUS) enables cross-sectional vessel evaluation, providing accurate external elastic membrane (EEM) based vessel sizing and detection of angiographically occult hematomas or severe dissections.^[7]^ Although IVUS-guided lesion preparation may facilitate optimized DCB deployment, real-world data confirming its long-term clinical efficacy in ACS remain scarce.

Previous studies have demonstrated that intravascular imaging-guided PCI facilitates procedural optimization by enabling accurate vessel sizing, assessment of plaque morphology, and detection of angiographically silent complications. These advantages may be particularly relevant for DCB angioplasty, in which procedural success depends largely on adequate lesion preparation, optimal balloon sizing, and homogeneous drug delivery without permanent scaffolding. However, evidence regarding the association between IVUS guidance and mid-term clinical outcomes in ACS patients undergoing DCB-only intervention remains limited.

Using inverse probability of treatment weighting (IPTW) to address baseline confounding, this single-center retrospective study evaluated the association between IVUS guidance, procedural characteristics, and clinical outcomes within a prespecified 24-month observation window in ACS patients treated with DCBs. The primary outcome measures were major adverse cardiovascular events (MACE) and net adverse clinical events (NACE).

## 2. Materials and methods

### 2.1. Study design and population

All archived clinical data were retrospectively reviewed after de-identification. The investigators did not have access to identifiable patient information.

This retrospective, single-center cohort study evaluated consecutive patients presenting with acute coronary syndrome (ACS) who underwent successful lesion preparation followed by drug-coated balloon (DCB) angioplasty at the First Affiliated Hospital of Shenzhen University from October 2017 to June 2025. Because this was a retrospective cohort study based on all eligible consecutive patients during the study period, the sample size was determined by the available patient population rather than by a prespecified power calculation. The study protocol was approved by the Institutional Review Board of the First Affiliated Hospital of Shenzhen University (Approval No. 2026-141-02PJ). Given the retrospective and de-identified nature of the analysis, the requirement for written informed consent was waived. All study procedures were conducted in accordance with the ethical principles outlined in the Declaration of Helsinki.

### 2.2. Inclusion criteria

Patients were included if they met the following criteria: a confirmed diagnosis of ACS, including unstable angina (UA), non-ST-segment elevation myocardial infarction (NSTEMI), or ST-segment elevation myocardial infarction (STEMI), according to the current guidelines^[8]^; the presence of a clinically significant target lesion, defined as >70% stenosis by visual estimation, or a fractional flow reserve (FFR) ≤0.80; successful lesion preparation prior to DCB deployment, strictly defined as residual stenosis <30%, Thrombolysis in Myocardial Infarction (TIMI) grade 3 flow, and the absence of National Heart, Lung, and Blood Institute (NHLBI) grade C or higher flow-limiting dissections; and the availability of complete procedural imaging and clinical follow-up information.

### 2.3. Exclusion criteria

Patients were excluded if they presented with any of the following: cardiogenic shock (Killip class IV), malignant arrhythmias, or a requirement for mechanical circulatory support upon admission; severe comorbidities significantly limiting life expectancy (e.g., advanced malignancy, end-stage renal or hepatic disease); known hypersensitivity or absolute contraindications to paclitaxel, contrast media, or dual antiplatelet therapy (DAPT); or highly complex target lesions necessitating rotational atherectomy, diffuse disease requiring planned stent implantations, or anatomical characteristics deemed otherwise unsuitable for a standalone DCB angioplasty.

### 2.4. Study groups and interventional strategies

Patients were categorized into 2 groups according to the intraprocedural imaging strategy used: IVUS-guided or angiography-guided intervention.

*Coronary angiography [CAG] group (n = 107):* Procedures were performed relying exclusively on standard 2-dimensional angiography with orthogonal projections. The reference vessel diameter (RVD) was determined by visual estimation to dictate the sizing for pre-dilation and the DCB, targeting a balloon-to-vessel ratio of 0.8 to 1.0.

*IVUS group (n = 122):* Procedures were guided by adjunctive IVUS imaging (Volcano systems, Philips Image Guided Therapy). IVUS was used to guide lesion assessment and procedural planning by precisely assessing the minimal lumen area (MLA) and the external elastic membrane (EEM).^[6]^ Consequently, DCB sizing was determined according to the EEM-derived RVD, targeting a balloon-to-vessel ratio of 0.9 to 1.0. Following DCB inflation, a final IVUS pullback was mandated to assess final vessel expansion and identify major dissections (NHLBI grade C or higher) or intramural hematomas, which would otherwise prompt bailout stenting.

The final analysis cohort comprised 229 ACS patients evaluated for baseline, procedural, and mid-term clinical outcomes. The decision to perform IVUS was made by the treating interventional cardiologists based on clinical judgment, lesion characteristics, anatomical complexity, and institutional practice patterns. IVUS utilization was not mandated by a predefined protocol during the study period.

### 2.5. Data extraction

Two independent investigators systematically extracted and verified all clinical and procedural data from the institutional electronic medical records and the Picture Archiving and Communication System (PACS). Any discrepancies were resolved by consensus or through arbitration by a third senior investigator. The extracted variables encompassed:

#### 2.5.1. Baseline clinical characteristics

Patient demographics, cardiovascular risk factors (hypertension, diabetes mellitus, smoking history, and family history of premature coronary artery disease), and prior coronary revascularization (percutaneous coronary intervention or coronary artery bypass grafting).

#### 2.5.2. Laboratory parameters

Admission blood samples (collected within 24 hours of presentation) were analyzed for biomarkers of myocardial injury (high-sensitivity cardiac troponin I [hs-cTnI]), ventricular stress (N-terminal pro-B-type natriuretic peptide [NT-proBNP]), renal function (creatinine), liver function (alanine aminotransferase [ALT], aspartate aminotransferase), coagulation profiles (thrombin time, activated partial thromboplastin time [APTT]), and cardiometabolic indices (low-density lipoprotein cholesterol [LDL-C], fasting blood glucose [FBG]).

#### 2.5.3. Clinical presentation and pharmacotherapy

The specific ACS classification (UA, NSTEMI, or STEMI) at index admission and details of the perioperative dual antiplatelet therapy (DAPT) regimens (aspirin combined with either clopidogrel or ticagrelor).

#### 2.5.4. Angiographic and procedural metrics

Arterial access site, target vessel location, lesion classification (de novo vs in-stent restenosis), and specific DCB parameters (quantity, diameter, and length) were recorded. Furthermore, any IVUS-driven modifications to the interventional strategy (e.g., additional lesion preparation or bailout stenting) were recorded.

### 2.6. Clinical endpoints and follow-up

#### 2.6.1. Follow-up procedures

Clinical follow-up was performed for 24 months after the index procedure, with clinical data primarily collected through routine outpatient visits and comprehensive reviews of institutional electronic medical records. Serial peripheral blood samples were collected during the 1- to 6-month and 6- to 24-month intervals after the procedure for systemic biochemical analyses. If multiple visits occurred within the observation window, data from the last visit during that window were used. In the present analysis, events occurring within the prespecified 24-month observation window were recorded and evaluated as cumulative binary outcomes, rather than time-to-event outcomes.

#### 2.6.2. Definitions of clinical endpoints

The primary clinical endpoint of this study was defined as major adverse cardiovascular events (MACE), a composite of cardiovascular death, target vessel or target lesion revascularization (TVR/TLR), and unplanned rehospitalization for recurrent acute coronary syndrome (ACS).^[9]^ Cardiovascular death and TVR/TLR events were adjudicated according to standardized criteria recommended by the Academic Research Consortium-2 (ARC-2) consensus document.^[10]^ To assess the overall balance between ischemic and bleeding outcomes, net adverse clinical events (NACE) were defined as the key secondary endpoint, consisting of MACE and major bleeding events. Additional secondary endpoints included the individual components of MACE and ACS-related rehospitalization analyzed separately.

#### 2.6.3. Safety outcomes and bleeding criteria

Acute procedural safety was evaluated based on the incidence of intra-procedural bailout stenting triggered by severe dissection or acute vessel closure. To systematically monitor follow-up biochemical profiles, comprehensive laboratory parameters – including biomarkers of myocardial injury, hepatic and renal function, fasting blood glucose, lipid profiles, and coagulation indices – were assessed at the 6 month and 24 month follow-up clinical visits. Furthermore, for the objective evaluation of NACE and hemorrhagic risk, major bleeding was rigorously defined as type 3 to 5 bleeding in accordance with the Bleeding Academic Research Consortium (BARC) consensus criteria.^[11]^

#### 2.6.4. Clinical event adjudication

To ensure the rigor and objectivity of outcome assessments, all suspected mid-term clinical events (inclusive of MACE, NACE, and bleeding episodes) were independently evaluated and adjudicated by 2 experienced cardiologists through a comprehensive review of inpatient medical records, imaging modalities, and follow-up clinical data. In the event of a discrepancy during the adjudication process, a senior physician was consulted to arbitrate and reach a final consensus.

### 2.7. Statistical analysis

All statistical analyses were performed using SPSS version 26.0 (IBM Corp., Armonk) and R software (version 4.2.1; R Foundation for Statistical Computing, Vienna, Austria). Propensity score stabilized inverse probability of treatment weighting (IPTW) was conducted with R; other conventional statistical analyses were completed in SPSS. All hypothesis tests were 2-tailed, and statistical significance was defined as *P <* .05. Subgroup interactions were assessed using weighted logistic regression models with the inclusion of an interaction term (e.g., treatment groups × clinical presentation status).

#### 2.7.1. Descriptive statistics

Continuous variables were assessed for normality using the Shapiro–Wilk test. Normally distributed data were described as mean* ± *standard deviation (SD) and were compared using the Student *t* test or Welch *t* test, as appropriate. Non-normally distributed data are presented as median with interquartile range (IQR) and were compared using the Mann–Whitney *U* test. Categorical variables are expressed as counts and percentages, with group comparisons performed utilizing the Pearson chi-square test or Fisher exact test, as clinically appropriate. Missing values in follow-up laboratory measurements were handled using multiple imputation by chained equations (MICE). To address the potential inflation of the type I error rate arising from multiple hypothesis testing across biochemical parameters assessed at multiple follow-up time points, these secondary laboratory analyses were considered exploratory, and nominal *P* values are reported without adjustment for multiple comparisons.

#### 2.7.2. Propensity score weighting (IPTW)

To rigorously adjust for potential baseline confounders and treatment selection bias between the 2 cohorts, inverse probability of treatment weighting (IPTW) was applied. The propensity scores were derived from a multivariable logistic regression model incorporating clinically relevant baseline clinical characteristics, clinical presentation, angiographic characteristics, and calendar year of PCI as a continuous covariate. These covariates included demographic characteristics, laboratory parameters, clinical presentations, angiographic and lesion characteristics, and baseline medications.

Post-weighting covariate balance was systematically evaluated via standardized mean differences (SMD), with an SMD < 0.10 strictly predefined as the threshold for indicating successful and optimal balancing between the groups.^[12,13]^ After weighting, the inter-group comparisons of clinical outcomes were performed using weighted logistic regression models, with treatment effects expressed as odds ratios (ORs) and corresponding 95% confidence intervals (CIs). Within this weighted framework, potential heterogeneity of the treatment effect across prespecified subgroups was assessed by introducing explicit multiplicative interaction terms (e.g., treatment group × clinical presentation status) into the regression models.

## 3. Results

### 3.1. Baseline and angiographic characteristics

The study cohort comprised 229 patients (IVUS-guided: n = 122; CAG-guided: n = 107; Table 1). Most baseline demographics and clinical covariates, including cardiovascular risk factors (hypertension, diabetes), prior PCI status, and renal/lipid profiles, did not differ statistically between the groups. Pre-procedural laboratory testing revealed a significant baseline disparity in WBC counts (*P* = .012), whereas hs-cTnI levels were comparable (*P* = .214). Notably, prominent baseline imbalances were observed in clinical presentation (Diagnosis: UA/NSTEMI/STEMI, *P* < .001) and the perioperative antiplatelet regimen, with a significantly higher proportion of patients receiving clopidogrel in the IVUS group than in the CAG group (76.2% vs 43.9%, *P* < .001).

**Table 1:** Comparison of baseline clinical characteristics and procedural characteristics between the 2 groups of patients.

Indicators (variables)	CAG (N = 107)	IVUS (N = 122)	*P*
Patient demographics and clinical profile			
Age (yr)	60.05 ± 12.84	58.83 ± 11.48	.324
Gender (male), n (%)			.185
Male	81 (75.7)	102 (83.6)	
Female	26 (24.3)	20 (16.4)	
Smoking history, n (%)			.702
No	42 (39.3)	52 (42.6)	
Yes	65 (60.7%)	70 (57.4%)	
Smoking age (yr)	30.0 (20.0, 40.0)	30.0 (15.0, 30.0)	.337
Family history (yes), n (%)			.458
No	86 (80.4)	92 (75.4)	
Yes	21 (19.6)	30 (24.6)	
History of hypertension, n (%)			.571
No	39 (36.4)	50 (41.0)	
Yes	68 (63.6)	72 (59.0)	
History of diabetes (%)			.323
No	67 (62.6)	85 (69.7)	
Yes	40 (37.4)	37 (30.3)	
PCI history, n (%)			1.000
No	82 (76.6)	94 (77.0)	
Yes	25 (23.4)	28 (23.0)	
Vital signs			
Systolic blood pressure (mm Hg)	139.02 ± 21.85	137.22 ± 22.00	.537
Diastolic blood pressure (mm Hg)	84.08 ± 13.97	85.57 ± 15.09	.480
Heart rate (beats/min)	76.0 (68.5, 86.0)	76.0 (71.0, 84.8)	.694
Laboratory tests (biochemistry and hematology)			
hs-CRP(mg/L)	2.03 (0.99, 5.93)	1.64 (0.58, 5.32)	.179
ALT (U/L)	31.0 (19.0, 40.0)	28.0 (19.1, 42.3)	.723
LDL-C (mmol/L)	2.59 (2.03, 3.32)	2.91 (2.01, 3.83)	.096
FBG (mmol/L)	6.20 (5.39, 7.82)	6.22 (5.34, 7.78)	.953
Scr (umol/L)	71.60 (62.75, 87.15)	73.35 (63.42, 83.90)	.795
K^+^ (mmol/L)	4.04 (3.80, 4.26)	4.06 (3.76, 4.25)	.644
WBC (10^9/L)	6.95 (5.87, 8.96)	8.02 (6.33, 9.86)	.012
Hb (g/L)	136.93 ± 16.22	139.51 ± 13.59	.420
PLT (10^9/L)	222.06 ± 52.99	223.57 ± 51.40	.826
APTT (s)	29.05 (27.02, 31.38)	29.40 (27.35, 31.25)	.555
d-dimer (mg/L)	0.28 (0.18, 0.46)	0.23 (0.16, 0.48)	.587
NT-proBNP (pg/ml)	240.0 (85.5, 646.0)	212.5 (73.0, 542.3)	.443
hs-cTnI (ng/ml)	0.10 (0.01, 1.18)	0.25 (0.02, 1.44)	.214
Procedural and lesion characteristics			
Diagnosis (UA/NSTEMI/STEMI)			<.001
UA, n (%)	46 (43.0)	23 (18.9)	
NSTEMI, n (%)	50 (46.7)	65 (53.3)	
STEMI, n (%)	11 (10.3)	34 (27.9)	
Lesion type, n (%)			.528
Type A/B	14 (13.1)	12 (9.8)	
Type C	93 (86.9)	110 (90.2)	
Degree of calcification			.815
Severe calcification, n (%)	30 (28)	37 (30.3)	
Non-severe calcification, n (%)	77 (72.0)	85 (69.7)	
Other			
DCB quantity	1.12 ± 0.35	1.15 ± 0.38	.536
DCB size (mm)	2.65 ± 0.38	2.78 ± 0.42	.15
Femoral artery access, n (%)			1.000
No	115 (93.5%)	100 (94.3%)	
Yes	7 (6.5%)	7 (5.7%)	
P2Y12 antagonist, n (%)			<.001
Ticagrelor	60 (56.1)	29 (23.8)	
Clopidogrel	47 (43.9%)	93 (76.2%)	
Bailout stenting, n (%)	3 (2.7%)	7 (5.5%)	.346
Vascular category, n (%)			.221
Major epicardial artery involvement, n (%)	73 (68.2%)	92 (75.4%)	
Branch blood vessels, n (%)	34 (31.8%)	30 (24.6%)	
Calendar year of PCI	2021 (2020–2023)	2024 (2022–2024)	<.001
Follow-up duration ≥6 months, n (%)	106 (99.1)	117 (95.9)	.219

Continuous variables are expressed as mean ± SD or median (IQR), and compared using the Student *t* test (or Welch *t* test) or the Mann–Whitney *U* test, as appropriate, depending on the data distribution. Categorical variables are presented as n (%) and compared utilizing the Pearson chi-square test or Fisher exact test.

ALT = alanine aminotransferase, APTT = activated partial thromboplastin time, CAG = coronary angiography, FBG = fasting blood glucose, Hb = hemoglobin, hs-CRP = high-sensitivity C-reactive protein, hs-cTnI = high-sensitivity cardiac troponin I, IQR = interquartile range, IVUS = intravascular ultrasound, LDL-C = low-density lipoprotein cholesterol, NSTEMI = non-ST-segment elevation myocardial infarction, NT-proBNP = N-terminal pro-B-type natriuretic peptide, PCI = percutaneous coronary intervention, PLT = platelet, Scr = serum creatinine, SD = standard deviation, STEMI = ST-segment elevation myocardial infarction, UA = unstable angina, WBC = white blood cell.

Regarding intra-procedural operations and lesion management strategies, the radial artery was the primary vascular access, and the requirement for femoral access was low and similar between groups (5.7% vs 6.5%, *P* = 1.000). Lesion characteristics, including American College of Cardiology/American Heart Association complexity (type C lesions, *P* = .528) and calcification severity (*P* = .815), were well matched. In terms of drug-coated balloon (DCB) utilization parameters, the mean number of DCBs used (1.15 ± 0.38 vs 1.12 ± 0.35, *P* = .536) and the immediate bailout stenting rates (5.5% vs 2.7%, *P *= .346) demonstrated high consistency between the IVUS and CAG groups. Notably, the mean diameter of the DCBs utilized in the IVUS group was slightly larger than that in the CAG group (2.78 ± 0.42 mm vs 2.65 ± 0.38 mm, *P* = .150). Although not statistically significant, this trend clinically reflects that IVUS assists operators in sizing balloons that more closely match the true vessel dimension to optimize drug-to-wall apposition.

### 3.2. IPTW adjustment and covariate balance

To eliminate the influence of the aforementioned baseline confounding factors (specifically WBC count, clinical diagnosis subtypes, P2Y12 inhibitor selection, and calendar year of PCI) on mid-term clinical outcomes, the inverse probability of treatment weighting (IPTW) method was employed. The evaluation of covariate balance following this adjustment is presented in Table 2. Notably, the standardized mean difference (SMD) for the calendar year of PCI was markedly reduced from 1.260 to 0.191, confirming that time-dependent bias was effectively minimized. The results demonstrated that after IPTW weighting, the standardized mean differences (SMD) for all adjusted covariates decreased significantly and remained below 0.1. These findings indicate that the baseline differences between the 2 cohorts were effectively eliminated, achieving statistical comparability and establishing a rigorous, unbiased foundation for the subsequent analysis of clinical endpoints within the prespecified 24-month observation window.

**Table 2:** Evaluation of the balance of baseline indicators between the pre- and post-weighting groups (comparison of SMDs across all indicators).

Indicators (variables)	SMD (before weighting)	Weighted SMD
Patient demographics and clinical profile		
Age (yr)	0.100	0.038
Gender (male), n (%)	0.196	0.019
Smoking history, n (%)	0.068	0.012
Smoking age (yr)	0.125	0.024
Family history (yes), n (%)	0.108	0.015
History of hypertension, n (%)	0.095	0.022
History of diabetes, n (%)	0.128	0.031
PCI history, n (%)	0.010	0.004
Vital signs		
Systolic blood pressure (mm Hg)	0.082	0.018
Diastolic blood pressure (mm Hg)	0.103	0.021
Heart rate (beats per minute)	0.052	0.011
Laboratory tests (biochemistry and hematology)		
hs-CRP (mg/L)	0.185	0.041
ALT (U/L)	0.065	0.014
LDL-C (mmol/L)	0.224	0.067
Scr (umol/L)	0.035	0.009
K^+^ (mmol/L)	0.041	0.008
WBC (10^9/L)	0.301	0.053
Hb (g/L)	0.172	0.035
PLT (10^9/L)	0.028	0.006
APTT (s)	0.076	0.021
d-dimer (mg/L)	0.088	0.019
NT-proBNP (pg/mL)	0.115	0.028
hs-cTnI (ng/mL)	0.142	0.033
Procedural and lesion characteristics		
Types of diagnosis (UA/NSTEMI/STEMI), n (%)	0.625	0.036
Vascular category, n (%)	0.160	0.013
Degree of calcification, n (%)	0.264	0.051
DCB quantity	0.082	0.015
DCB average diameter (mm)	0.315	0.042
Femoral artery access, n (%)	0.128	0.032
Calendar year of PCI	1.260	0.191
Clopidogrel (P2Y12 antagonist) use, n (%)	0.696	0.049

ALT = alanine aminotransferase, APTT = activated partial thromboplastin time, DCB = drug-coated balloon, FBG = fasting blood glucose, Hb = hemoglobin, hs-CRP = high-sensitivity C-reactive protein, hs-cTnI = high-sensitivity cardiac troponin I, IQR = interquartile range, LDL-C = low-density lipoprotein cholesterol, NSTEMI = non-ST-segment elevation myocardial infarction, NT-proBNP = N-terminal pro-B-type natriuretic peptide, PCI = percutaneous coronary intervention, PLT = platelet, Scr = serum creatinine, SD = standard deviation, SMD = standardized mean difference, STEMI = ST-segment elevation myocardial infarction, UA = unstable angina, WBC = white blood cell.

### 3.3. Primary clinical endpoint and safety outcomes

The proportion of patients with ≥6 months of available follow-up was comparable between the IVUS and CAG groups (95.9% vs 99.1%, *P* = .135). Within the prespecified 24-month observation window, the incidence of MACE was lower in the IVUS-guided group than in the CAG-guided group (8.2% vs 17.8%; *P =* .030). After IPTW adjustment, IVUS guidance remained associated with a lower incidence of MACE (OR: 0.41; 95% CI: 0.18–0.93; IPTW *P =* .038).

Similarly, the incidence of NACE was significantly lower in the IVUS-guided group compared with the CAG-guided group (9.8% vs 26.2%; IPTW *P* = .001), with an IPTW-adjusted OR of 0.31 (95% CI: 0.15–0.64).

### 3.4. Detailed analysis of secondary endpoints

We performed a detailed evaluation of individual clinical components to identify the main drivers of the overall benefit observed in the IVUS cohort (Table 3).

**Table 3:** Clinical outcomes and procedural efficiency metrics after IPTW adjustment.

Indicators (variables)	CAG (n = 107)	IVUS (n = 122)	Unadjusted OR (95% CI)	Statistical values (*t*/χ^2^)	Original *P* -value	IPTW *P* -value
Primary clinical endpoint						
MACE, n (%)*	19 (17.8)	10 (8.2)	0.41 (0.18–0.93)	4.771	.030	.038
Primary clinical endpoint						
NACE, n (%)†	28 (26.2)	12 (9.8)	0.31 (0.15–0.64)	10.547	.002	.001
Secondary endpoint						
ACS-related rehospitalization, n (%)	21 (19.6)	8 (6.6)	0.29 (0.12–0.68)	8.81	.005	.048
Bleeding events, n (%)	12 (11.2)	2 (1.6)	0.13 (0.03–0.60)	9.47	.004	.015
TVR/TLR, n (%)	2 (1.9)	3 (2.5)	1.32 (0.22–8.07)		1.000	.849
Cardiovascular mortality, n (%)	1 (0.9)	1 (0.8)	0.88 (0.05–14.18)		1.000	.965
Bailout stenting, n (%)	3 (2.8)	7 (5.5)	2.11 (0.53–8.37)	1.95	.346	.887
Health economics indicators						
Total length of stay (d)	5.77 ± 2.51	4.20 ± 1.87	–	−5.27	<.001	<.001
Post-procedural length of stay (d)	3.46 ± 1.81	3.36 ± 1.33	–	−0.47	.644	.658
Procedural duration (min)	106.82 ± 121.13	88.08 ± 25.12	–	−1.56	.119	.424

*Data presentation:* Continuous variables are expressed as the mean* ± *standard deviation (mean ± SD); comparisons between groups were performed using an independent samples *t* test. Categorical variables are presented as the number of cases and percentage, n (%); comparisons between groups were performed using the chi-square test or Fisher exact test. For variables with small cell counts evaluated by Fisher exact test, no chi-square values are reported. Adjustments were made for all baseline covariates detailed in Methods Section 2.7.2. –, Not applicable. Odds ratios (ORs) and 95% confidence intervals (CIs) were calculated for binary clinical endpoints only and are not applicable to continuous variables.

ACS = acute coronary syndrome, CAG = coronary angiography, CI = confidence interval, IPTW = inverse probability of treatment weighting, IQR = interquartile range, IVUS = intravascular ultrasound, MACE = major adverse cardiovascular events, NACE = net adverse clinical events, OR = odds ratio, SD = standard deviation, TLR = target lesion revascularization, TVR = target vessel revascularization.

* MACE: Defined as a composite endpoint comprising cardiovascular death, readmission for ACS, and target vessel/target lesion revascularization (TVR/TLR).

† NACE: Defined as a composite endpoint of MACE and major bleeding events.

#### 3.4.1. ACS-related rehospitalization

The incidence of unplanned rehospitalizations driven by recurrent ACS was markedly attenuated in the IVUS-guided arm compared with the CAG group (6.6% vs 19.6%; unadjusted *P =* .005). This benefit remained statistically significant after comprehensive covariate adjustment (IPTW *P =* .048).

#### 3.4.2. Bleeding events

Likewise, major bleeding events were substantially reduced in the IVUS group (1.6% vs 11.2%; unadjusted *P =* .004), and this protective effect was sustained in the weighted cohort (IPTW *P =* .015).

#### 3.4.3. Hard endpoints and procedural safety

In contrast, hard clinical endpoints were rare and evenly distributed between the 2 groups. Rates of cardiovascular mortality (0.8% vs 0.9%; unadjusted *P =* 1.000) and target vessel or target lesion revascularization (TVR/TLR; 2.5% vs 1.9%; unadjusted *P =* 1.000) showed no significant differences, either before or after baseline adjustment (all IPTW *P >* .05). Regarding procedural safety, the need for bailout stenting did not differ significantly between groups in the unweighted analysis (5.5% vs 2.8%; unadjusted *P =* .346). Although a slight numerical trend emerged after IPTW adjustment, it did not reach statistical significance (IPTW *P >* .05).

### 3.5. Procedural efficiency and hospitalization metrics

We also evaluated operational efficiency and resource utilization. The overall hospitalization duration was significantly shorter in the IVUS-guided group than in the conventional CAG group (4.20* ± *1.87 days vs 5.77* ± *2.51 days; *P <* .001). In contrast, procedure‑related time metrics – specifically, mean procedural time (88.08* ± *25.12 minutes vs 106.82* ± *121.13 minutes) and post-procedural length of stay – did not differ significantly between the 2 groups (both *P >* .05).

### 3.6. Prespecified subgroup analysis

Subgroup analyses evaluated the consistency of IVUS-derived clinical benefit across clinical and anatomical subsets (Table 4). Significant effect modification was observed for patient diagnosis (*P*_for interaction_ = .020). In contrast, lesion location showed no significant heterogeneity (*P*_for interaction_ = .621), while the degree of calcification exhibited a borderline interaction trend (*P*_for interaction_ = .074).

**Table 4:** Subgroup analysis.

Subgroup Characteristics	CAG (n = 107) MACE, n (%)	IVUS (n = 122) MACE, n (%)	OR (95% CI)	*P*	Interaction P*
General population	19 (17.8%)	10 (8.2%)	0.41 (0.18–0.93)	.03	
Diagnosis					.020
UA, n (%)	13 (28.3%)	1 (4.3%)	0.13 (0.03–0.56)	.006	
AMI, n (%)	6 (9.8%)	9 (9.1%)	0.93 (0.43–2.01)	.846	
Degree of calcification					.074
Non-severe calcification, n (%)	10 (13.0%)	7 (8.2%)	0.62 (0.30–1.29)	.202	
Severe calcification, n (%)	9 (30.0%)	3 (8.1%)	0.20 (0.07–0.55)	.002	
Lesion location					.621
Main branch lesions, n (%)	13 (17.8%)	8 (8.7%)	0.44 (0.21–0.90)	.024	
Non-main branch lesions, n (%)	6 (17.6%)	2 (6.7%)	0.34 (0.08–1.55)	.165	

AMI = acute myocardial infarction, CAG = coronary angiography, CI = confidence interval, IVUS = intravascular ultrasound, MACE = major adverse cardiovascular events, OR = odds ratio, UA = unstable angina.

* The smaller the *P*_for interaction_, the greater the influence of this factor on the clinical benefit of IVUS.

#### 3.6.1. Lesion location

Within the stratum of main branch lesions, IVUS guidance was associated with a significant reduction in MACE (8.7% vs 17.8%; OR: 0.44; 95% CI: 0.21–0.90; *P =* .024). A consistent, though statistically nonsignificant, trend was noted in non-main branch lesions (6.7% vs 17.6%; *P =* .165; *P*_for interaction_ = .621).

#### 3.6.2. Calcification severity

For target lesions complicated by heavy calcification, the incidence of MACE was profoundly curtailed in the IVUS group compared to the conventional cohort (8.1% vs 30.0%; OR: 0.20; 95% CI: 0.07–0.55; *P =* .002). The interaction test across calcification severity strata reached a borderline threshold (*P*_for interaction_ = .074), suggesting a possible differential association that requires confirmation in larger studies.

#### 3.6.3. Clinical presentation

Driven by the significant interaction (*P*_for interaction_ = .020), further stratification revealed pronounced IVUS benefit in patients with unstable angina (4.3% vs 28.3%; OR: 0.13; 95% CI: 0.03–0.56; *P =* .006). In contrast, MACE rates were comparable between groups in patients presenting with acute myocardial infarction (AMI) (9.1% vs 9.8%; OR: 0.93; 95% CI: 0.43–2.01; *P =* .846).

### 3.7. Biochemical profiles during predefined follow-up windows

To characterize biochemical profiles after DCB angioplasty, systemic laboratory parameters were assessed during the follow-up windows of 1 to 6 months and 6 to 24 months after the procedure (Table 5). In the overall cohort, all assessed biochemical parameters, including cardiac injury markers, renal function, hepatic function, metabolic profiles, and coagulation indices, showed no significant differences between the IVUS-guided and CAG-guided groups during follow-up.

**Table 5:** Comparison of biochemical profiles between IVUS-guided and angiography-guided DCB angioplasty groups during the 1 to 6 months and prespecified 24-month observation windows.

Indicators	1–6 mo CAG	1–6 mo IVUS	*P* (1–6 mo)	6–24 mo CAG	6–24 mo IVUS	*P* (6–24 mo)
Myocardial injury biomarkers						
NT-proBNP (pg/mL)	138.2 ± 62.5	126.4 ± 45.8	.180	784.3 ± 3190.7	422.3 ± 634.1	.650
hs-cTnI (ng/mL)	0.015 ± 0.008	0.012 ± 0.005	.269	0.31 ± 0.92	1.33 ± 3.14	.452
Renal function and metabolic profile						
Scr (µmol/L)	84.32 ± 22.2	82.15 ± 18.4	.685	89.62 ± 40.3	82.37 ± 21.5	.621
FBG (mmol/L)	6.95 ± 1.88	7.12 ± 2.15	.612	6.98 ± 2.53	7.69 ± 2.93	.223
LDL-C (mmol/L)	1.88 ± 0.75	1.81 ± 0.82	.562	1.87 ± 0.76	1.81 ± 0.91	.423
Liver function and coagulation						
ALT (U/L)	26.84 ± 15.3	24.52 ± 12.6	.209	33.34 ± 19.1	33.22 ± 16.8	.785
APTT (S)	31.85 ± 5.12	32.41 ± 4.25	.372	29.31 ± 4.03	29.36 ± 4.82	.394

Values are presented as mean ± standard deviation or median (interquartile range), as appropriate. Biochemical markers were compared between the IVUS-guided and CAG-guided groups during the 1- to 6-month and 6- to 24-month observation windows after the procedure. Continuous variables were compared using the Student *t* test (or Welch *t* test) or the Mann–Whitney *U* test, as appropriate, according to data distribution. A 2-sided *P* value < .05 was considered statistically significant. Missing values were handled using multiple imputation by chained equations.

ALT = alanine aminotransferase, APTT = activated partial thromboplastin time, CAG = coronary angiography, DCB = drug-coated balloon, FBG = fasting blood glucose, hs-cTnI = high-sensitivity cardiac troponin I, IPTW = inverse probability of treatment weighting, IVUS = intravascular ultrasound, LDL-C = low-density lipoprotein cholesterol, NT-proBNP = N-terminal pro-B-type natriuretic peptide, Scr = serum creatinine.

#### 3.7.1. Cardiac biomarkers

No significant intergroup differences were observed in NT-proBNP or hs-cTnI levels among available measurements between 1 and 6 months after DCB angioplasty (NT-proBNP: 126.4 ±* *45.8 vs 138.2 ± 62.5 pg/mL, *P =* .180; hs-cTnI: 0.012* ± *0.005 vs 0.015* ± *0.008 ng/mL, *P =* .269), or between 6 and 24 months follow-up window (NT-proBNP: *P =* .650; hs-cTnI: *P =* .452).

#### 3.7.2. Renal and metabolic profiles

Renal clearance capacity was robustly preserved across both study groups. Serum creatinine measurements indicated no temporal or intergroup variations at either 1 to 6 months (82.15* *±* *18.4 vs 84.32* *± 22.2 μmol/L; *P =* .685) or 2 years (82.37* ± *21.5 vs 89.62* ± *40.3 μmol/L; *P =* .621). Parallel trends were observed in core metabolic parameters, with fasting glucose and LDL-C trajectories remaining comparable between the cohorts throughout the observational window (all *P >* .05).

#### 3.7.3. Hepatic and coagulation indices

Hepatic function and coagulation parameters were consistent between cohorts. No significant disparities were detected in ALT (24.52* ± *12.6 vs 26.84* ± *15.3 U/L; *P =* .209) or APTT (32.41* ± *4.25 vs 31.85* ± *5.12 seconds; *P =* .372) among available measurements between 1 and 6 months after DCB angioplasty. Similar findings were observed between 6 and 24 months after DCB angioplasty (ALT: *P =* .785; APTT: *P =* .394).

## 4. Discussion

### 4.1. Core findings and net clinical benefit

Our findings demonstrated that IVUS guidance was associated with a lower incidence of MACE and NACE within the prespecified 24-month observation window in patients with ACS undergoing DCB angioplasty. Compared to angiography guidance alone, IVUS was associated with a marked reduction in the primary endpoint of MACE (8.2% vs 17.8%; IPTW *P =* .038). Importantly, NACE – which integrates both procedural safety and clinical efficacy – was substantially lower in the IVUS cohort (9.8% vs 26.2%; IPTW *P <* .001). These findings suggest that IVUS-guided lesion optimization may contribute to improved net clinical outcomes in ACS patients undergoing DCB angioplasty.^[7,14]^

### 4.2. Potential mechanisms underlying the observed reduction in MACE

The observed reduction in MACE, which was mainly attributable to a lower incidence of ACS-related rehospitalization (IPTW *P =* .048), may reflect the potential contribution of IVUS-guided lesion optimization. Angiography alone is limited by its 2-dimensional projection, often failing to identify angiographically silent but clinically significant occult dissections (e.g., grade C or higher) or intramural hematomas. Consequently, the numerically higher rate of bailout stenting in the IVUS cohort (6.6% vs 2.8%) likely reflects the enhanced detection of severe, flow-limiting defects rather than representing procedural failure alone.^[15]^

The universal benefit of intravascular imaging in complex PCI was recently challenged by the IVUS-CHIP and OPTIMAL trials (NEJM, 2026).^[16,17]^ Both studies reported no prognostic advantage of IVUS over angiography for high-risk stenting in European cohorts. In contrast, our findings suggest that IVUS guidance may be associated with a lower incidence of MACE components during the prespecified 24-month observation window after DCB therapy. This difference may be explained by the potential advantages of IVUS-guided lesion assessment, vessel sizing, and procedural optimization. In DES implantation, imaging serves primarily as an adjunct for post-stent optimization. For DCB angioplasty, however, it is a prerequisite for assessing plaque morphology and ensuring adequate lesion preparation prior to drug delivery. Additionally, consistent with the RENOVATE-COMPLEX-PCI trial,^[18,19]^ our findings suggest the prognostic yield of IVUS-guided interventions may be more pronounced in populations with clinical characteristics similar to those enrolled in the present study.

By confirming adequate luminal gain, assessing residual plaque burden, and ruling out severe elastic recoil, IVUS eliminates unrecognized anatomical vulnerabilities. This precise morphological optimization facilitates superior vessel healing, thereby mitigating the risk of late target vessel thrombosis and recurrent MACE components.^[7]^

### 4.3. Target populations: main branches, heavy calcification, and unstable angina

Subgroup analyses showed that IVUS-guided DCB therapy was associated with enhanced patient benefit in specific lesion locations, lesion characteristics, and diagnostic types. While DCBs have conventionally been indicated for small vessel disease, the present study observed a lower MACE rate in the IVUS‑guided group when these devices were applied to main branch lesions (8.7% vs 17.8%).^[20]^ Given the larger myocardial territory at risk, optimizing luminal gain in these vessels is paramount. IVUS allows for accurate 1:1 balloon-to-artery sizing, thereby mitigating the risk of flow-limiting dissections that often result from angiographic misjudgment.

Extending the “leave nothing behind” approach to large epicardial arteries represents a shift in PCI strategy. While DCBs were initially favored in small de novo lesions to avoid the high failure rates of small-diameter stents, their rationale in large main branches extends beyond restenosis prevention. The goal is to preserve physiological vasomotion, allow for late lumen enlargement, and eliminate the risk of very late stent thrombosis or neo-atherosclerosis.^[20]^ However, treating large vessels with a DCB-only approach requires rigorous lesion preparation. Because angiography alone frequently underestimates vessel caliber and plaque compliance, IVUS is highly valuable to ensure adequate plaque modification while avoiding severe dissection, thereby safely expanding the applicability of DCBs to larger and more complex anatomies.^[14]^

Irrespective of the degree of coronary calcification, the clinical benefit derived from IVUS guidance remained consistent (*P*_for interaction_ = .074). Standard 2-dimensional angiography has well-known limitations in assessing plaque compliance and identifying calcium fracture. IVUS, by contrast, provides real-time visualization of calcium distribution and confirms therapeutic plaque modification. This step is critical to ensure uniform drug delivery and adequate luminal expansion in heavily calcified lesions.^[7]^

In addition, a significant interaction was observed across different clinical presentation subgroups (*P*_for interaction_ = .020), wherein patients with unstable angina (UA) derived a more pronounced clinical association than those with AMI. This difference likely reflects distinct pathophysiological mechanisms. The heavy thrombus burden associated with AMI can obscure imaging details and complicate accurate vessel sizing. Conversely, UA lesions frequently feature lipid-rich, vulnerable plaques that are highly susceptible to longitudinal plaque shift and edge dissection after balloon inflation.^[21,22]^ In these cases, IVUS is instrumental in quantifying plaque burden and identifying relatively healthy reference segments to serve as safe “landing zones.” This targeted approach minimizes edge-related complications and subsequent target lesion failure.^[23]^

### 4.4. Synergistic safety gains and health economic value

IVUS guidance was associated with lower rates of ischemic and bleeding-related outcomes (1.6% vs 11.2%; IPTW *P =* .015). This observed safety benefit likely stems from improved anatomical assessment. In standard DCB angioplasty, the risk of acute vessel closure from flow-limiting dissections or intramural hematomas often necessitates prolonged, high-intensity antithrombotic therapy. Routine IVUS use confirms optimal luminal expansion and excludes severe dissection, securing a stable “stent-like” outcome.^[1]^ This definitive anatomical feedback allows operators to safely de-escalate or shorten post-procedural antithrombotic regimens, thereby lowering bleeding risk without sacrificing ischemic protection. Although the retrospective design precludes eliminating all confounding related to individualized antithrombotic prescriptions, this mechanism provides a clinically plausible explanation for how IVUS optimizes the balance between bleeding and ischemia.^[8]^

Beyond clinical outcomes, the upfront cost of IVUS catheters is likely offset by downstream resource conservation. IVUS guidance significantly reduced total hospital length of stay (4.20* ± *1.87 days vs 5.77* ± *2.51 days; *P <* .001), reflecting fewer perioperative events and abbreviated post-procedural observation.^[24]^ By accelerating discharge and minimizing adverse outcomes, IVUS presents a favorable health-economic profile that may support further evaluation of its cost-effectiveness within DCB workflows.

### 4.5. Serial biochemical profiles during follow-up

While primary cardiovascular endpoints remain the standard measure of PCI success, the systemic impact of these interventional procedures warrants rigorous longitudinal evaluation. Although IVUS has traditionally been associated with theoretical risks of prolonged procedure duration and increased contrast load that could compromise renal function, our biochemical follow‑up during the 1- to 6-month and 6- to 24-month periods after DCB angioplasty showed no significant intergroup differences in the assessed biochemical parameters, based on available measurements. These findings provide no evidence of clinically meaningful differences in systemic biochemical profiles between the groups.^[25]^

During longitudinal observation, core systemic parameters – including cardiac biomarkers (NT-proBNP, hs-cTnI), hepatorenal indices (ALT, creatinine), and metabolic profiles – remained stable and highly comparable between the cohorts throughout the available follow-up period. These data provide reassurance that the use of IVUS-guided lesion assessment in combination with local DCB drug delivery was not associated with adverse changes in systemic biochemical profiles or end-organ function during follow-up.^[26]^

### 4.6. Limitations

Several limitations should be acknowledged. The retrospective, single-center, and non-randomized design of this study may have introduced selection bias. Although IPTW was used to reduce imbalance between groups, residual confounding from unmeasured factors cannot be fully ruled out. In particular, some detailed intravascular morphological features, including plaque rupture and thrombus burden, were not systematically available before IVUS assessment and therefore were not included in the propensity score model.

Second, clinical outcomes were analyzed as cumulative binary events within the prespecified 24-month observation window rather than using time-to-event analyses. Therefore, potential differences in the timing of clinical events during follow-up could not be evaluated. The relatively small sample size (n = 229) may have limited the statistical power of the study, especially for subgroup analyses involving specific clinical presentations or lesion characteristics. Additionally, there were temporal disparities in the length of available follow-up between the treatment strategies. While the calendar year of PCI was balanced using the IPTW model and outcomes were framed within a prespecified 24-month window, the unequal availability of late-term observational data might have contributed to the under-ascertainment of long-term clinical events.

In addition, IVUS images were analyzed by local investigators rather than an independent core laboratory, which may have introduced assessment variability and limited the consistency of quantitative morphological measurements. Detailed information on the duration of dual antiplatelet therapy (DAPT) after discharge was unavailable, preventing a direct assessment of whether differences in DAPT duration contributed to the observed bleeding outcomes.

Finally, although procedure-related parameters and length of hospital stay were evaluated, a formal cost-effectiveness analysis was not performed. Further multicenter prospective studies with larger populations are needed to confirm the clinical impact and potential economic value of IVUS-guided DCB angioplasty in patients with ACS.

## 5. Conclusion

In patients with acute coronary syndrome undergoing drug-coated balloon angioplasty, IVUS guidance was associated with a lower incidence of MACE and NACE within the prespecified 24-month observation window compared with angiography guidance. These findings suggest a potential association with improved clinical outcomes of IVUS-guided lesion optimization in ACS patients treated with a “leaving nothing behind” strategy, although prospective studies are required for confirmation. This association was generally consistent across several clinically relevant subgroups, including main branch and heavily calcified lesions, with a stronger association observed among patients presenting with unstable angina. Furthermore, IVUS guidance was associated with lower rates of ischemic and bleeding-related outcomes without clinically relevant deterioration in routinely measured biochemical parameters or end-organ function during follow-up. These findings support further evaluation of IVUS-guided lesion preparation as a strategy to optimize DCB angioplasty in ACS patients.

## Acknowledgments

The authors would like to thank the staff of the Department of Cardiology and the Digital Subtraction Angiography (DSA) Room at the First Affiliated Hospital of Shenzhen University for their assistance in data collection and procedural support.

## Author contributions

**Conceptualization:** Wei Wen.

**Data curation:** Wei Wen.

**Formal analysis:** Wei Wen.

**Methodology:** Dongdong Yu.

**Supervision:** Haibo Chen.

**Validation:** Dongdong Yu.

**Writing – original draft:** Wei Wen.

**Writing – review & editing:** Haibo Chen.
